# Sex-Specific Metabolic Footprint of Ketogenic Diet in C57BL/6J Mice

**DOI:** 10.3390/biomedicines14020462

**Published:** 2026-02-19

**Authors:** Marko Sablić, Viktoria Čurila, Barbara Viljetić, Lovro Mihajlović, Zeljka Korade, Károly Mirnics, Irena Labak, Leonarda Murvaj, Senka Blažetić, Vedrana Ivić, Željko Debeljak, Marta Balog, Marija Heffer

**Affiliations:** 1 Department of Anatomy and Neuroscience, Faculty of Medicine Osijek, Josip Juraj Strossmayer University of Osijek, 31000 Osijek, Croatia; msablic@mefos.hr; 2 Department of Medical Biology and Genetics, Faculty of Medicine Osijek, Josip Juraj Strossmayer University of Osijek, 31000 Osijek, Croatia; 3 Department of Medicinal Chemistry, Biochemistry and Clinical Chemistry, Faculty of Medicine Osijek, Josip Juraj Strossmayer University of Osijek, 31000 Osijek, Croatia; 4 Department of Biology, Josip Juraj Strossmayer University of Osijek, 31000 Osijek, Croatia; 5 Department of Pediatrics, University of Nebraska Medical Center, Child Health Research Institute, Omaha, NE 68198, USA; 6 Munroe-Meyer Institute for Genetics and Rehabilitation, University of Nebraska Medical Center, Child Health Research Institute, Omaha, NE 68198, USA; 7 Clinical Institute of Laboratory Diagnostics, University Hospital Centre Osijek, 31000 Osijek, Croatia; 8 Department of Pharmacology, Faculty of Medicine Osijek, Josip Juraj Strossmayer University of Osijek, 31000 Osijek, Croatia

**Keywords:** ketogenic diet, oxysterols, skeletal muscle, metabolome

## Abstract

**Background/Objectives**: The ketogenic diet (KD) induces profound metabolic shifts, yet the sex-specific long-term effects on skeletal muscle metabolism and sterol homeostasis across tissues remain insufficiently characterized. This study tested the hypothesis that a prolonged KD would elicit distinct, sex-dependent metabolic and sterol adaptations in mice. **Methods**: We examined how a 12-week KD, compared with a standard diet, affected body mass, the skeletal muscle metabolome, hepatic lipid and collagen content, and sterol profiles in the skeletal muscle, liver, spleen, and serum in male and female C57BL/6J mice. Three-month-old mice of both sexes were randomized to a KD or standard diet and evaluated using the histological quantification of hepatic steatosis and collagen deposition, matrix-assisted laser desorption/ionization time-of-flight imaging mass spectrometry (MALDI-TOF IMS) of skeletal muscle, and LC-MS/MS-based sterol profiling. **Results**: The KD induced rapid body mass gain in males and delayed weight gain in females, promoted hepatic steatosis in both sexes, and generated clearly segregated, sex- and diet-specific skeletal muscle metabolomic signatures. These signatures included reduced tricarboxylic acid cycle precursors and a marked decrease in S-adenosylmethionine in KD-fed females. Across tissues, the KD consistently suppressed precursor sterols, including 7-dehydrocholesterol and desmosterol in the skeletal muscle, liver, and spleen, while elevating serum cholesterol and desmosterol (male-biased), with changes generally more pronounced in males. **Conclusions**: Collectively, these findings demonstrate that a long-term KD drives sex- and organ-specific metabolic remodeling, with evidence of greater metabolic flexibility but a shared risk of hepatic steatosis in females. These results underscore the importance of personalized, sex-stratified approaches when considering long-term ketogenic interventions.

## 1. Introduction

Dietary composition is a major determinant of systemic metabolism and tissue-specific physiological adaptation. Among dietary interventions, the ketogenic diet (KD)—characterized by high fat, moderate protein, and very low carbohydrate intake—induces a profound metabolic shift from glucose utilization toward fatty acid oxidation and ketone body production [1,2]. Originally developed as a therapeutic strategy for drug-resistant epilepsy [3,4], the KD is gaining increasing attention for its broad metabolic effects, influencing energy homeostasis, lipid metabolism, and cellular signaling across multiple organ systems [5]. Indeed, numerous studies have demonstrated the efficacy of the KD in improving insulin sensitivity, reducing systemic inflammation, and promoting weight loss, making it a popular tool for managing metabolic syndrome and obesity [6].

The liver plays a central role in systemic metabolic adaptation to the KD by coordinating fatty acid uptake, β-oxidation, and ketone body production [7]. Although the KD can promote efficient lipid utilization and metabolic flexibility, its hepatic effects are strongly context-dependent. A short-term KD or KD applied in models of nonalcoholic fatty liver disease or diabetes often reduces hepatic steatosis through enhanced β-oxidation and improved insulin sensitivity, whereas prolonged KD feeding in healthy rodents, including C57BL/6J mice, has been shown to induce hepatic lipid accumulation, as observed in the present study. This temporal duality, together with the sex-specific responses identified here, highlights the metabolic complexity of the KD. In susceptible individuals, prolonged ketogenic feeding may promote hepatic steatosis and extracellular matrix remodeling, with distinct responses in males and females that depend on the dietary composition and duration [8]. Consistent with this view, recent clinical and mechanistic studies indicate that extended or unrestricted ketogenic feeding can drive adverse metabolic remodeling, including hepatic lipid accumulation and dyslipidemia, in a manner influenced by biological sex [9]. Experimental evidence further suggests that females generally exhibit more efficient hepatic lipid handling and reduced susceptibility to maladaptive remodeling compared with males, underscoring the sex-specific mechanisms of hepatic adaptation to dietary stress [10].

Beyond the liver, skeletal muscle is a major regulator of whole-body energy homeostasis due to its substantial capacity for substrate utilization, mitochondrial energy production, and fatty acid oxidation. In response to carbohydrate restriction, skeletal muscle enhances lipid-derived fuel utilization and undergoes mitochondrial metabolic reprogramming to sustain energy production during prolonged ketosis [11,12,13]. Importantly, skeletal muscle metabolism exhibits pronounced sex-specific differences. Variations in sex hormone signaling, mitochondrial efficiency, and lipid handling pathways contribute to divergent metabolic responses in males and females under both physiological and dietary stress conditions [14,15]. Despite the growing interest in ketogenic nutrition, comprehensive analyses of sex-dependent skeletal muscle adaptation to a prolonged KD remain limited, restricting our ability to define sex-specific metabolic trajectories and predict long-term physiological outcomes.

In addition to classical metabolic organs, the spleen represents a key immune organ whose function is tightly regulated by the membrane lipid composition and cholesterol availability. Sterols and their biosynthetic intermediates modulate lipid raft organization and immune cell activation, thereby linking cholesterol metabolism with systemic immunometabolic adaptation [16,17]. Because dietary fat intake and ketogenic metabolism profoundly reshape lipid availability, the spleen constitutes a relevant peripheral tissue for evaluating diet-induced sterol remodeling [18]. In this context, sterol homeostasis remains a critical yet comparatively underexplored component of ketogenic adaptation. Cholesterol and its biosynthetic intermediates, including lanosterol, desmosterol, and dehydrocholesterols, are key regulators of lipid raft organization, intracellular signaling, and mitochondrial function [18,19]. Perturbations in sterol metabolism can influence membrane dynamics, redox balance, and inflammatory signaling, thereby affecting tissue function under metabolic stress [20]. Nevertheless, systematic investigations addressing how long-term ketogenic feeding reshapes sterol profiles across circulation and metabolically distinct tissues in a sex-dependent manner remain scarce [21].

Together, these metabolic and sterol-regulating pathways form an integrated network supporting the metabolic flexibility and high energy efficiency induced by ketogenic nutrition. While KDs have demonstrated broad metabolic benefits across diverse physiological contexts, emerging evidence suggests that long-term ketogenic adaptations are not uniform and may vary substantially according to biological sex, tissue-specific programming, and sterol homeostasis. Integrating skeletal muscle spatial metabolomics with multi-organ sterol profiling offers a powerful framework for capturing both adaptive and potentially maladaptive responses to prolonged ketogenic feeding. To date, no studies have systematically profiled sterol intermediates across serum, the liver, the spleen, and skeletal muscle in a sex-stratified manner under a prolonged KD, leaving a critical gap in our understanding of tissue-specific cholesterol homeostasis.

Accordingly, we sought to characterize diet- and sex-dependent metabolic and sterol adaptations to 12-week ketogenic feeding in C57BL/6J mice, with a focus on skeletal muscle metabolomics (MALDI-TOF IMS), liver histology, and sterol profiling across the serum, spleen, liver, and muscle. By integrating spatial metabolomics, histological analysis, and targeted LC-MS/MS-based sterol quantification across sexes and tissues, this study demonstrates (i) the detection of unanticipated sex × diet × tissue interactions, (ii) mechanistic links between muscle metabolism and systemic sterol homeostasis, and (iii) evidence for personalized KD risk stratification.

We hypothesized that prolonged KD feeding would elicit distinct, sex-dependent metabolic remodeling and alterations in sterol profiles across tissues, with males showing greater susceptibility to hepatic steatosis and females displaying enhanced skeletal muscle metabolic flexibility, consistent with known sex differences in lipid handling and hormone-modulated substrate utilization.

## 2. Materials and Methods

### 2.1. Animal Model and Study Design

**Figure 1:**
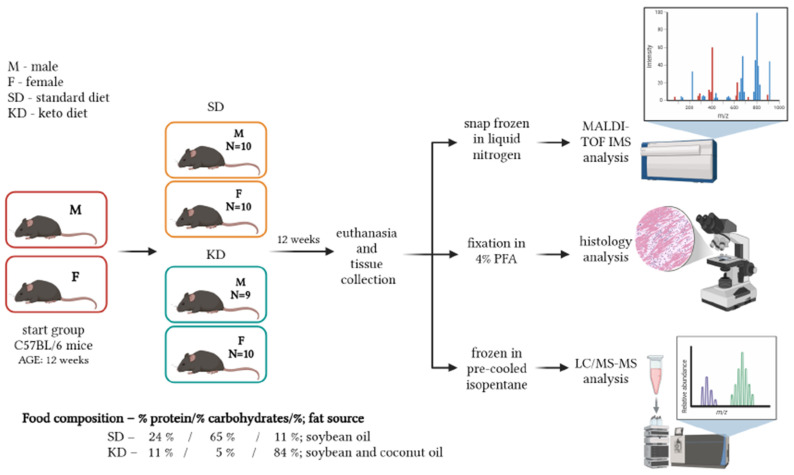
Experimental flowchart. Created in BioRender, with permission. Balog, M. (2026) https://BioRender.com/18baynd (accessed on 10 February 2026) [26].

The study included 39 three-month-old C57BL/6J mice of both sexes—19 males (M) and 20 females (F)—from our in-house breeding colony (registration number HR-POK-005). The number of animals (sample size) was determined using the G*Power software (version 3.1.2, University of Kiel, Kiel, Germany) and GraphPad Prism (version 10.6.1; GraphPad Software, San Diego, CA, USA). Calculations were based on a one-way ANOVA with power of 0.8, an effect size (*f*) of 0.8, and an α level of 0.05 across four equally sized groups. While the analysis indicated a minimum of 32 animals (*n* = 8 per group), additional subjects were included to account for potential attrition. Three-month-old mice modeled young-adult KD users; this age captures metabolic flexibility before age-related declines. Mice were randomly assigned to either the control group, which was fed a standard chow diet (SD) (Altromin Spezialfutter GmbH & Co., Lage, Germany; C1324), consisting of 10 males (SDM) and 10 females (SDF), or the experimental group, which was on the KD (Altromin Spezialfutter GmbH & Co., Lage, Germany; C1084), consisting of 9 males (KDM) and 10 females (KDF). Treatment lasted 12 weeks. The final sample size for the KDM group reflected the natural distribution of sex and age within the available litters. Priority was given to using littermates to minimize interindividual variability, which resulted in a slightly smaller cohort for the KDM group due to the stochastic nature of in-house breeding. The KD consisted of 84% fat (with coconut oil and soybean as fat sources), 11% protein, and 5% carbohydrates, whereas the SD contained 11% fat (soybean as the fat source), 24% protein, and 65% carbohydrates (Figure 1). Animals were group-housed (up to 5 animals per cage) in self-ventilating cages (EHRET, Freiburg, Germany) with five air changes per minute, under controlled environmental conditions: temperature 20–23 °C, relative humidity 40–60%, and a 12 h light/12 h dark cycle. Food and water were provided ad libitum. Body mass was measured weekly (every morning on the first day of each experimental week) using a digital scale (SPX621, Ohaus Corp., Parsippany, NJ, USA). At the end of the 12-week feeding period, animals were euthanized by cervical dislocation. Food intake was not quantified, as ad libitum feeding was chosen to mimic voluntary KD adherence in humans without imposed calorie restrictions, with body mass changes providing a direct measure of cumulative energy balance outcomes. Blood and tissue samples were collected immediately after euthanasia. For serum collection, blood samples were centrifuged at 3000 rpm for 10 min (Eppendorf 5804; Hamburg, Germany). The obtained serum was mixed with butylated hydroxytoluene (BHT) (Sigma-Aldrich, St. Louis, MO, USA) and triphenylphosphine (TPP) (Sigma-Aldrich, St. Louis, MO, USA) solution and stored at −80 °C until further analysis. For histological examination, liver and skeletal muscle samples were fixed in 4% paraformaldehyde (PFA) (Acros Organics, Geel, Belgium). Skeletal muscle samples intended for MALDI-TOF IMS analysis were freshly frozen in liquid nitrogen and stored at −80 °C. Snap-frozen samples of liver, spleen, and skeletal muscle in pre-cooled isopentane (Fisher Chemical, Göteborg, Sweden) were stored at −80 °C for sterol analysis using LC-MS/MS. The animal study was approved by the Croatian Ministry of Agriculture on 31 October 2022 (class: UP/I-322-01/22-01/22, registration number: 525-09/566-22-4). All experimental procedures were conducted in accordance with the European Communities Council Directive (86/609/EEC) and national regulations on the protection of animals used for scientific purposes.

### 2.2. Histological Staining

Liver tissues were sliced into 25 µm thick cross-sections using a cryostat (CM3050S, Leica, Nussloch, Germany). For histological evaluation, a randomly selected representative subset of *n* = 5 animals per group was used. Three non-consecutive sections were collected per animal to ensure representative sampling of the hepatic tissue. Lipid droplets were visualized using Oil Red O staining. General morphology was assessed using hematoxylin–eosin (HE) staining [22], and collagen content in the liver was assessed using Picrosirius red staining. All stainings were performed according to standard histological protocols [23]. Sections were imaged using a light microscope (Carl Zeiss, Axioskop 2 MOT, Jena, Germany) equipped with a digital camera (Olympus DP70, Olympus Optical, Japan) at 20× objective magnification. Quantification of lipid droplets was performed using FIJI (version 1.53; National Institutes of Health, Bethesda, MD, USA) [24], following the methodology previously published by our group [25]. Briefly, a standardized region of interest of 250 μm × 250 μm was defined for each microphotograph. Images were converted to an 8-bit grayscale format and inverted so that higher numerical values represented higher staining intensities. Each hepatic section was imaged at five randomly selected fields of view, resulting in a total of 15 images per biological replicate. The integrated density (IntDen) was measured by a researcher blinded to group assignments. To account for intra-animal variability, ensure biological relevance, and avoid pseudo-replication, IntDen values from all 15 images per animal were averaged to yield a single representative value per biological replicate for subsequent statistical analysis.

### 2.3. MALDI-TOF IMS Skeletal Muscle Analysis

Matrix-assisted laser desorption/ionization time-of-flight imaging mass spectrometry (MALDI-TOF IMS) analysis was performed using the Shimadzu iMScope TRIO MALDI-IT-TOF instrument (Shimadzu, Kyoto, Japan) after daily calibration with 2,5-dihydroxybenzoic acid (DHB; Sigma-Aldrich, St. Louis, MO, USA). Fresh-frozen skeletal muscle tissues from SDM, SDF, KDM, and KDF mice (*n* = 9–10 per group) were cryosectioned at 16 μm thickness and mounted on indium tin oxide (ITO)-coated glass slides with surface resistivity of 15–25 Ω/sq (Sigma-Aldrich, St. Louis, MO, USA). After three washes with 20 mM ammonium acetate buffer (Merck, Darmstadt, Germany), the sections were dried and immediately processed further. The matrix α-cyano-4-hydroxycinnamic acid (CHCA) (Sigma-Aldrich, St. Louis, MO, USA) was applied using an iMLayer sublimation device (Shimadzu, Kyoto, Japan) according to the manufacturer’s instructions (10 min sublimation at 250 °C). Sublimation was followed by 2 min of recrystallization at 64.7 °C using 500 μL of 0.5% methanol (Thermo Fisher Scientific, Waltham, MA, USA). Imaging in positive ion mode was conducted over an *m*/*z* range of 300–700 Da with the following parameters: 36 pixels with a 10 × 10 μm pitch, a laser diameter of 10 μm (D1), a laser intensity of 21.7%, 50 laser shots, and a laser frequency of 200 Hz. IMS data visualization was performed using ImageReveal, version 1.1.010128 (Shimadzu, Kyoto, Japan). Tentative metabolite assignments were derived through partial least squares discriminant analysis (PLS-DA) in conjunction with the Human Metabolome Database (HMDB) [27] and the METASPACE [28] database, applying an *m*/*z* tolerance of 50 ppm, positive polarity, a MALDI ionization source, and “mouse” as the organism. Only *m*/*z* signals with an intensity of at least 10% of the highest measured value were analyzed. The remaining *m*/*z* ratios produced signals that were too weak and were therefore excluded from the analysis. To assess the classification accuracy, 10-fold cross-validation repeated 100 times was used. Variations in signal intensities across experimental groups were illustrated using box and whisker plots.

### 2.4. Sterol Analysis

Sterol profiling was carried out following derivatization with 4-phenyl-1,2,4-triazoline-3,5-dione (PTAD), according to previously published procedures [29]. The processed extracts were analyzed using an Acquity UPLC system (Waters, Milford, MA, USA) fitted with an ANSI-standard well plate holder and interfaced to a Thermo Scientific TSQ Quantis triple-quadrupole mass spectrometer (Thermo Fisher Scientific, Waltham, MA, USA) equipped with an atmospheric pressure chemical ionization (APCI) source. A 10 µL aliquot of each sample was injected onto a Phenomenex Luna Omega C18 column (1.6 µm, 100 Å, 2.1 × 100 mm). Chromatographic separation was achieved using a mobile phase of methanol (90%) and acetonitrile (10%) containing 0.1% (*v*/*v*) acetic acid, with a flow rate of 0.5 mL/min and a total runtime of 1.7 min. The detection of native sterols was performed in selective reaction monitoring (SRM) mode, monitoring the transitions *m*/*z* 369 → 369 for cholesterol (CHOL), *m*/*z* 560 → 365 for 7-dehydrocholesterol (7-DHC), *m*/*z* 592 → 560 for desmosterol (DES), and *m*/*z* 634 → 602 for lanosterol (LAN), with retention times of 0.7, 0.4, 0.3, and 0.3 min, respectively. Internal standards were analyzed using the SRM transitions *m*/*z* 376 → 376 for d_7_-cholesterol, *m*/*z* 567 → 372 for d_7_-7-DHC, *m*/*z* 595 → 563 for ^13^C_3_-desmosterol, and *m*/*z* 637 → 605 for ^13^C_3_-lanosterol.

### 2.5. Statistical Analysis

All statistical analyses were performed using the Prism software, version 10.6.1 (GraphPad Software LLC, Boston, MA, USA), IBM SPSS Statistics (version 29.0.2.0; IBM Corp., 2023) [30], and R software (version 4.4.1; R Foundation for Statistical Computing, Vienna, Austria) [31]. Numerical data are presented as the mean ± standard deviation for normally distributed variables or as the median with interquartile range for non-normally distributed variables. Data normality was assessed using the Shapiro–Wilk test, while the heterogeneity of variance was evaluated via Brown–Forsythe test.

Differences in body mass, and the quantification of Oil Red O and Picosirius red stainings, were analyzed using one-way ANOVA followed by Tukey’s post hoc test, provided that the data met the assumptions of normality and homogeneity of variance. In instances of heteroscedasticity, Welch’s ANOVA with Dunnett’s T3 post hoc test was applied. Non-normally distributed data were assessed via the Kruskal–Wallis test followed by Dunn’s multiple-comparison post hoc test. A *p*-value < 0.05 was considered statistically significant.

To evaluate the robustness of our histological findings, effect sizes were quantified using Cohen’s *d*. For lipid accumulation (ORO), an exceptionally large effect size (*d* = 4.3) was observed, confirming that our sample size provided statistical power exceeding 0.99. In contrast, the collagen content (PSR) showed a small-to-moderate effect size (*d* ≈ 1.8), which would necessitate a substantially larger cohort (*n* = 18–25) to achieve statistical significance. Consequently, the PSR data were interpreted as the biological absence of pathological fibrosis under the current experimental conditions, rather than a result of insufficient statistical power.

MALDI-TOF IMS data were processed and analyzed within the R statistical environment. Multivariate and univariate analyses, including partial least squares discriminant analysis (PLS-DA), the construction of network plots, and the analysis of the classification accuracy, were conducted using the mixOmics package 6.32.0 [29].

Prior to interpreting the ANOVA results, the equality of variances across groups was assessed using Levene’s test; since the assumption was met for all analyzed variables (*p* > 0.05), the ANOVA results were deemed valid. Serum sterol levels were analyzed using a two-way ANOVA, whereas organ sterol data were assessed via repeated-measures ANOVA. In the repeated-measures model, organ was treated as a within-subjects factor, while sex and diet were included as between-subjects factors. Sphericity was evaluated using Mauchly’s test, and when it was violated, Greenhouse–Geisser corrections were applied. For both models, a post hoc analysis was performed using pairwise comparisons based on estimated marginal means, with the Bonferroni correction applied for multiple comparisons. Effect sizes for each main effect and interaction are reported as the partial eta squared (η_p_^2^), representing the proportion of variance in the dependent variable explained by each factor. Statistical significance was set at *p* ≤ 0.05.

## 3. Results

The physiological impact of a 12-week ketogenic diet (KD) was evaluated through a multi-modal approach, integrating tissue morphology, spatial metabolomics, and targeted sterol quantification in male and female C57BL/6J mice.

### 3.1. Body Mass

**Figure 2:**
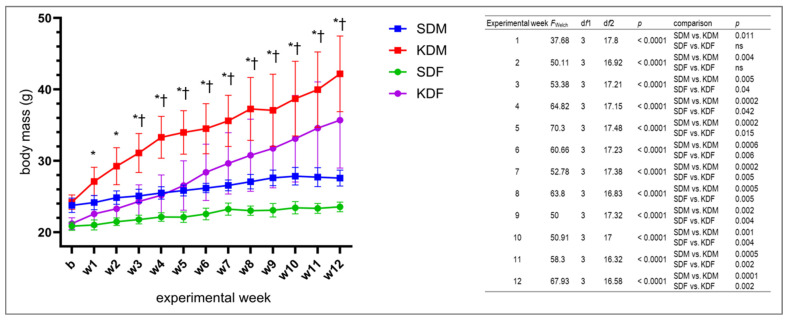
Body mass of male (M) and female (F) mice over 12 weeks on a standard diet (SD) or ketogenic diet (KD). Data are presented as mean ± standard deviation. Body mass was recorded at baseline (*b*) and weekly (*w*1–*w*12). Statistical significance was assessed using Welch’s one-way ANOVA followed by Dunnett’s T3 multiple-comparisons post hoc test to account for heteroscedasticity. * *p* < 0.05 for SDM vs. KDM; † *p* < 0.05 for SDF vs. KDF. *n* = 10 per group, except KDM, where *n* = 9. A summary of Welch’s *F* statistics and exact *p*-values is provided in the table (**right**), ns—not significant.

Body mass changed in a sex- and diet-dependent manner over the 12-week intervention (Figure 2). KD-fed males (KDM) exhibited a rapid increase in body mass, with significant differences relative to SDM detectable as early as week 1; this divergence continued to widen over subsequent weeks. In contrast, KD-fed females (KDF) displayed a delayed onset of weight gain, with significant differences relative to standard diet females (SDF) emerging at week 3. Despite these distinct temporal patterns, both KD groups ultimately achieved comparable cumulative percentage increases in body mass, converging from approximately week 9 onward (Figure S1). In contrast, SDM and SDF exhibited minimal changes in body mass throughout the study.

### 3.2. Histological Alterations in Liver Tissue Induced by Ketogenic Diet

#### 3.2.1. Ketogenic Diet Triggers Pronounced Hepatic Lipid Deposition

**Figure 3:**
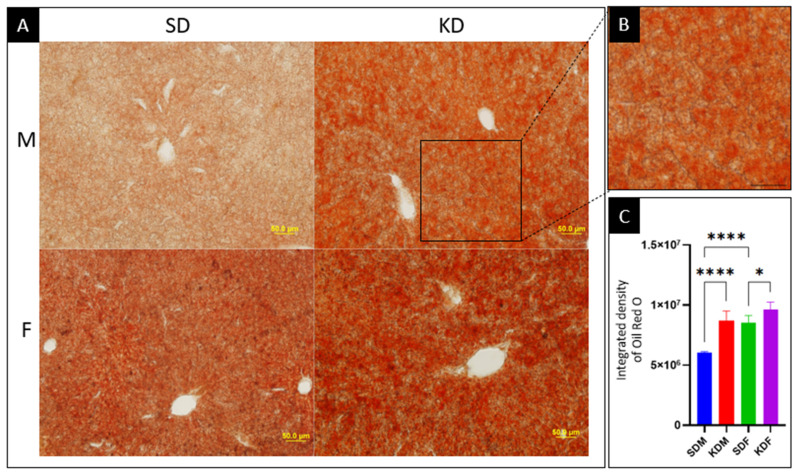
The ketogenic diet induces sex-specific hepatic lipid accumulation. (**A**) Representative micrographs of Oil Red O-stained liver sections from male (M) and female (F) mice after 12 weeks on a standard (SD) or ketogenic diet (KD). The images show the distribution of neutral lipids within the hepatic lobules, mainly localized around the central veins. Scale bar: 50 μm. (**B**) Inset showing a high-magnification view of a representative region of interest (ROI; area of 0.06 mm^2^) used for the quantification of lipid droplets (**C**) Quantitative analysis of Oil Red O integrated density (*n* = 5). Data are presented as the mean ± SD of the integrated staining intensity, with higher values indicating increased lipid content. Statistical significance (one-way ANOVA followed by Tukey’s post hoc test): * *p* < 0.05, **** *p* < 0.0001.

Hepatic lipid droplet accumulation was quantified by Oil Red O staining (Figure 3). The one-way ANOVA revealed significant group differences (*p* < 0.001; *F* (3,16) = 33.04; *R*^2^ = 0.86). Post hoc Tukey analysis showed that KD-fed males exhibited a marked increase in staining intensity compared with SDM (*p* < 0.001), corresponding to a 43.29% relative increase. Although SDF exhibited a higher baseline staining intensity than SDM (*p* < 0.001), females demonstrated a substantially attenuated diet-induced response, with KDF showing only a 12.62% relative increase compared with SDF.

Overall, females displayed higher Oil Red O staining intensities across both diets, indicating greater baseline hepatic lipid content. However, diet-induced lipid accumulation was significantly more pronounced in males, revealing a sex-specific susceptibility to KD-induced steatosis despite lower initial hepatic lipid levels.

#### 3.2.2. Ketogenic Diet Did Not Affect Hepatic Collagen Content

**Figure 4:**
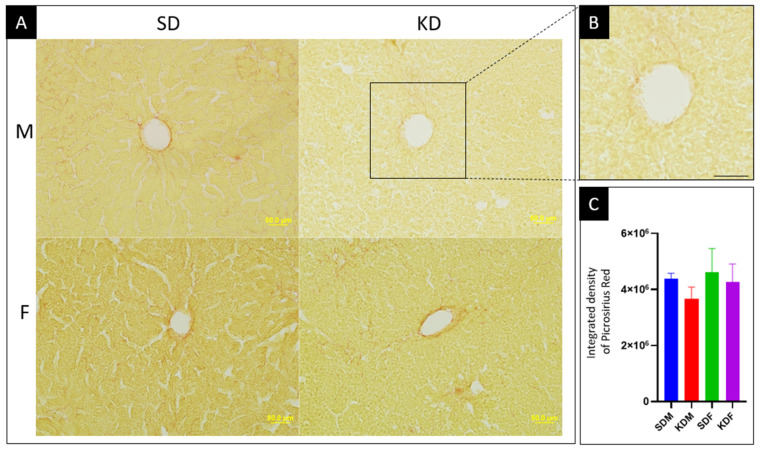
Hepatic collagen distribution. (**A**) Representative micrographs of Picrosirius red-stained liver sections from male (M) and female (F) mice after 12 weeks on a standard (SD) or ketogenic diet (KD). Bright red staining indicates collagen fibers primarily localized in the adventitia of the central veins. Scale bar: 50 μm. (**B**) High-magnification inset of a representative hepatic lobule used for connective tissue assessment. Scale bar: 50 μm. (**C**) Quantitative analysis of Picrosirius red integrated density (*n* = 5 for all animal groups). Data are presented as the mean ± SD of the integrated staining intensity, with higher values indicating increased collagen content. No statistically significant differences were observed (one-way ANOVA, *p* = 0.11, *F* (3, 16) = 2.4, *R*^2^ = 0.31).

Hepatic collagen content was assessed using Picrosirius red staining (Figure 4). In contrast to the pronounced lipid accumulation, 12 weeks of the KD did not induce hepatic fibrosis. A modest, non-significant reduction in collagen density was observed in KD-fed males (16.5% decrease; *p* > 0.05), whereas females maintained stable collagen levels across diets. These findings indicate the preservation of the hepatic extracellular matrix integrity despite sustained ketogenic metabolic stress at this time point.

### 3.3. MALDI-TOF IMS Results

**Figure 5:**
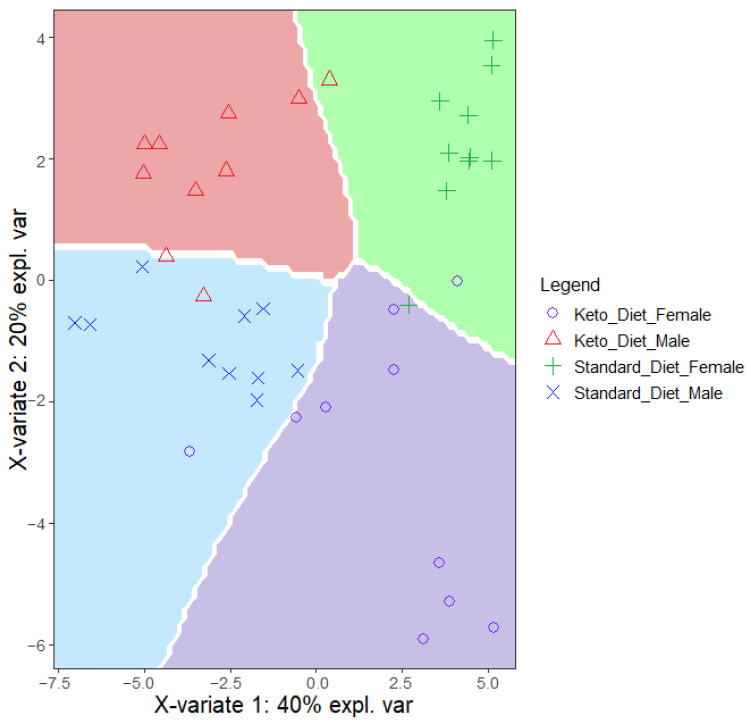
Partial least squares discriminant analysis (PLS-DA) classification of skeletal muscle samples. Dataset heterogeneity is shown across the four experimental groups: standard diet males (SDM) (blue area), standard diet females (SDF) (green area), ketogenic diet males (KDM) (red area), and ketogenic diet females (KDF) (purple area). *n* = 10 for all groups, except KDM, where *n* = 9.

MALDI-TOF IMS analysis was performed on skeletal muscle tissue from the four experimental groups, SDM, SDF, KDM, and KDF, within a mass range of 300–700 Da (average spectra shown in the Supplementary Materials). PLS-DA of the full spectral dataset effectively separated samples by diet and sex (Figure 5). Dataset heterogeneity was lowest in the SDF group, as reflected by the smallest variance envelope (green region in Figure 5).

**Figure 6:**
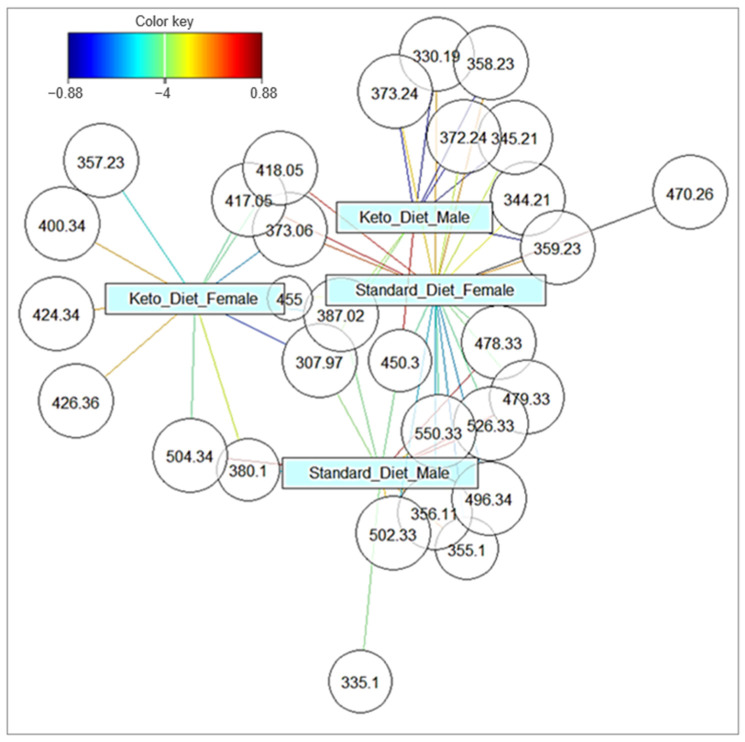
Network plot of *m*/*z* signal distribution in skeletal muscle across the different animal groups. The *m*/*z* quotients present in the two groups suggest metabolic similarity between these groups. The color key that correlates with the significance of the connectivity is shown in the upper-left corner of the figure, with a red color indicating a strong connection (i.e., the specificity of the *m*/*z* signal for a certain group) and a blue color indicating a weak connection. *n* for all groups = 10, except for KDM, where *n* = 9.

The classification accuracy reached 80% when using the first two principal components and exceeded 95% with five components (Figures S2 and S3). Notably, a receiver operating characteristic (ROC) analysis based on the first two components yielded high areas under the curve (AUC = 0.87–0.99) across all groups (Figure S4), indicating robust group discrimination with minimal model complexity. The distribution of significantly altered *m*/*z* features between groups is illustrated as a network plot in Figure 6. Approximately half of the discriminating *m*/*z* features were group-specific, while the remainder were shared between two groups.

Table 1 lists tentatively annotated single-hit *m*/*z* signals, highlighting those with significantly altered levels in KDM and KDF compared to SDM and SDF. These features were selected due to having the highest loadings in the PLS-DA model, ensuring that the reported metabolites represented the primary drivers of metabolic divergence between groups.

**Figure 7:**
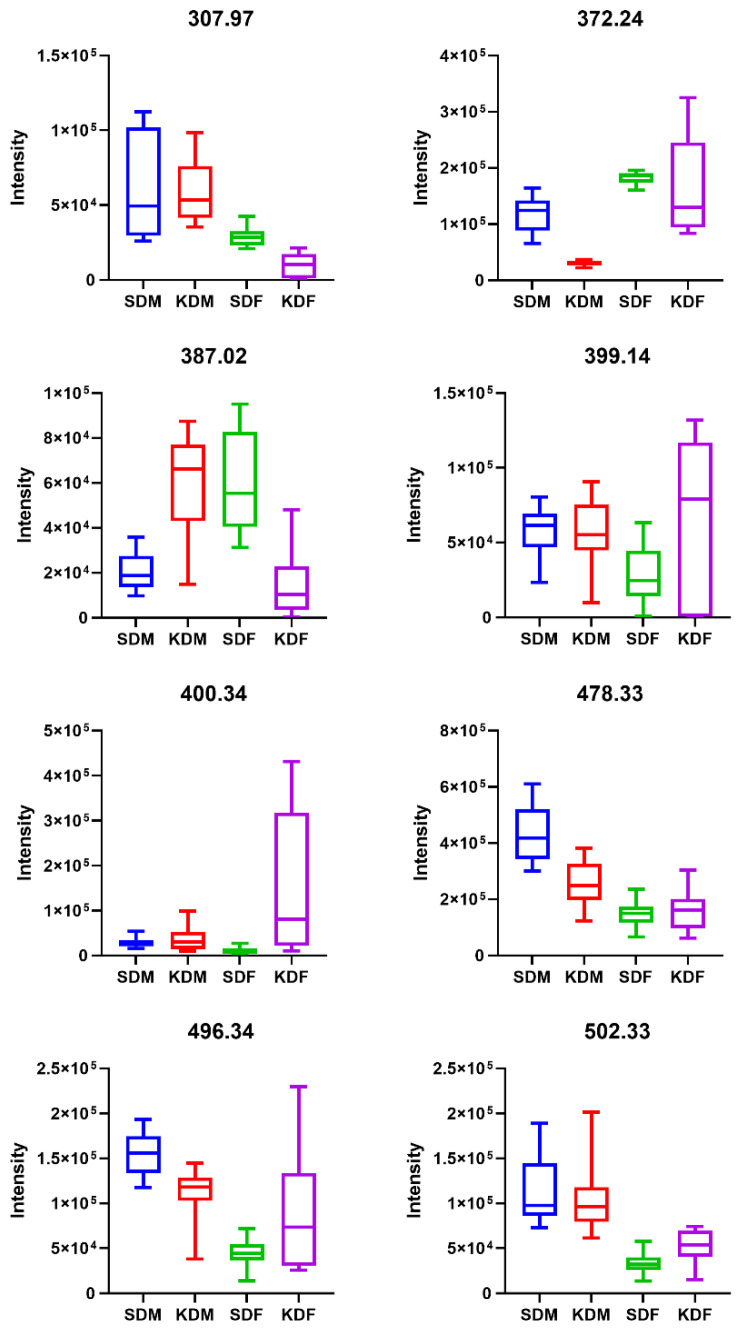
Skeletal muscle *m*/*z* signal intensities across experimental groups. Box and whisker plots show intensities for key discriminative *m*/*z* values. These signals were identified as unique “single-hit” matches in lipid databases; however, they are presented with their *m*/*z* values to maintain technical precision. Groups: standard diet males (SDM; blue), ketogenic diet males (KDM; red), standard diet females (SDF; green), and ketogenic diet females (KDF; purple). *n* = 10 for all groups, except KDM, where *n* = 9.

The comparison of the tentatively annotated single-hit *m*/*z* signal intensities across the four groups (SDM, KDM, SDF, KDF) is shown using box and whisker plots (Figure 7).

**Figure 8:**
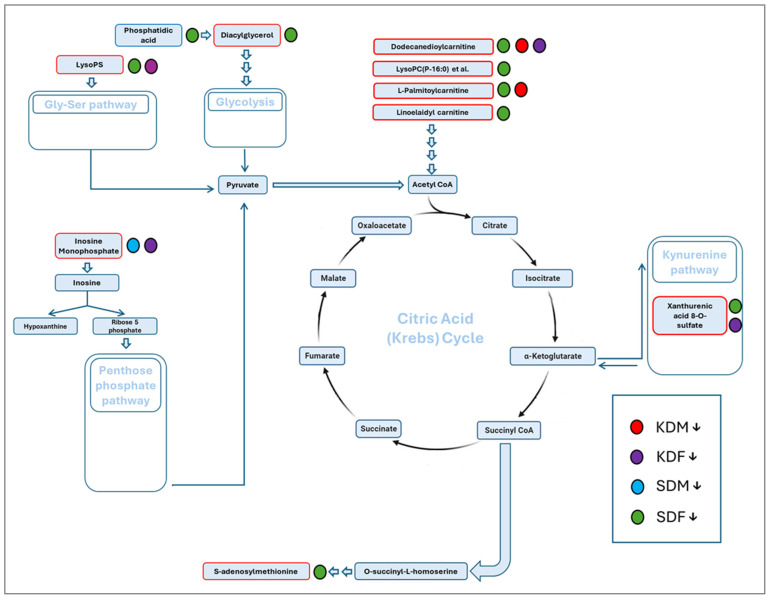
Schematic representation of MALDI-TOF tentative metabolite hits in relation to the Krebs cycle. The diagram shows tentatively annotated metabolic entry points into the tricarboxylic acid (TCA or Krebs) cycle, highlighting metabolites that were significantly decreased in each treatment group compared to all other experimental groups, based on a 4 × 4 contingency analysis (this approach identifies group-specific metabolic suppression patterns, as detailed in Table S1). Tentatively annotated metabolites are positioned according to their biochemical pathways, feeding into β-oxidation, anaplerotic reactions, amino acid catabolism, or direct TCA intermediates. Color coding indicates the treatment group in which each metabolite was reduced: blue for standard diet males (SDM), red for ketogenic diet males (KDM), green for standard diet females (SDF), and purple for ketogenic diet females (KDF).

Distinct treatment-specific metabolic suppression patterns were observed (Figure 8). SDF exhibited the most extensive downregulation of lipid-derived intermediates, converging on acetyl-CoA, whereas the remaining groups showed fewer overall reductions. This schematic representation illustrates how diet- and sex-specific decreases affect distinct biochemical entry points into the tricarboxylic acid cycle, revealing divergent metabolic remodeling across groups.

### 3.4. Organ-Specific Sterol Content Analysis in Mice Treated with Ketogenic Diet

**Figure 9:**
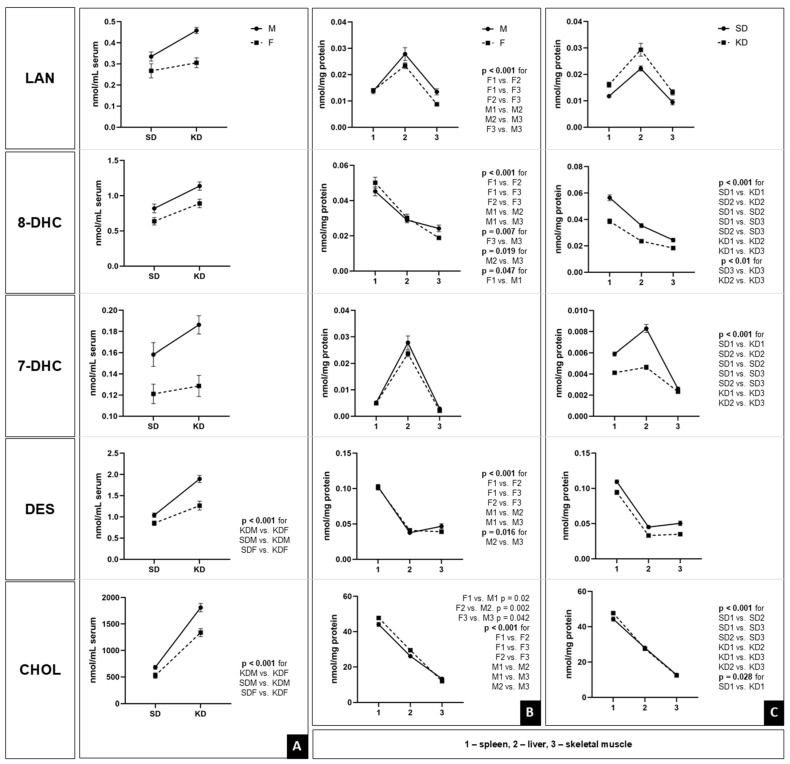
Comparative analysis of sterol profiles in serum and various organs under different sex and dietary conditions. (**A**) Serum levels: concentrations of lanosterol (LAN), 8-dehydroxycholesterol (8-DHC), 7-dehydroxycholesterol (7-DHC), desmosterol (DES), and cholesterol (CHOL) in the serum of male (M) and female (F) subjects on standard (SD) and ketogenic diets (KD). Statistical analysis was performed using two-way ANOVA, highlighting the pronounced sexual dimorphism and dietary responsiveness of CHOL and DES. (**B**) Organ × sex interaction: sterol distribution across the spleen (1), liver (2), and skeletal muscle (3) as a function of sex (M vs. F). (**C**) Organ × diet interaction: sterol distribution across the organs as a function of diet (SD vs. KD). Data in panels (**B**,**C**) were analyzed using repeated-measures ANOVA followed by Bonferroni-corrected pairwise comparisons based on estimated marginal means (EMM). All data are presented as EMM ± SEM. *n* = 10 for all groups, except KDM, where *n* = 9. Numbers on the x-axis correspond to 1—spleen, 2—liver, and 3—skeletal muscle. Statistical significance and effect sizes (partial eta squared) are detailed in the accompanying tables. Sterol concentrations in organs were normalized to protein content to ensure comparability across different tissues.

The distribution of analyzed sterols in the serum and organs (spleen, liver, and skeletal muscle) is shown in Figure 9. Statistical significance and effect sizes are provided in the accompanying Table 2 (serum) and Table 3 (organs). The sterols are presented in order of their sequential positions in the CHOL biosynthetic pathway, from the initial precursor, LAN to the final product, CHOL.

#### 3.4.1. Sterol Profile Analysis in Serum

All analyzed serum sterols showed a significant dependence on sex, while the influence of diet and its interaction with sex varied depending on the specific sterol. In the earlier steps of the biosynthetic pathway, the effects of sex and diet were additive, without significant interactions (Table 2).

The levels of LAN were significantly affected by sex and diet, which acted independently. Males had higher concentrations than females, and the KD groups showed elevated levels of this sterol regardless of sex (males > females; KD > SD). Similarly to LAN, significant effects of sex and diet were observed for 8-DHC, with both factors acting independently. The level of 7-DHC was influenced exclusively by sex, and the KD did not cause significant changes in its levels. The model explained 43.1% of the variance, indicating that serum 7-DHC levels remain stable with respect to diet and are primarily determined by sex. In contrast to the early and intermediate precursors of CHOL, a strong sex × diet interaction was found for DES and CHOL, suggesting that the KD does not affect these sterols equally in males and females. Significant main effects on DES levels were found for sex and diet, along with a significant interaction. Post hoc (Bonferroni) analysis showed that while concentrations were similar between sexes in the SD, the KD caused a dramatic increase that was nearly twice as great in males (an increase of approximately 0.85 nmol/mL) compared to females (approximately 0.41 nmol/mL). CHOL showed the most pronounced changes in serum. In addition to the main effects, the sex × diet interaction was highly significant. Although the KD drastically increased the CHOL levels in both sexes (*p* < 0.001), the increase was substantially larger in males (approximately +1126 mg/dL) than in females (approximately +812 mg/dL). Notably, the effect of sex became dominant in the KD group, whereas it was not significant in the SD group.

#### 3.4.2. Sterol Profile Analysis in Organs

Sterol concentrations in the spleen, liver, and skeletal muscle were primarily determined by the specific organ, while dietary and sex-related factors showed varying degrees of influence across the biosynthetic pathway (Table 3).

Across all five sterols, the organ was the most powerful determinant, with exceptionally large effect sizes ranging from 0.76 to 0.97. These results indicate that sterol localization accounts for nearly all observed variability, reflecting highly specialized metabolic profiles for each tissue.

For intermediate precursors (7-DHC, 8-DHC, and DES), the KD was the primary driver of change, while sex played a minimal or secondary role. Significant main effects on 7-DHC concentrations were found for both organ and diet. However, a significant organ × diet interaction showed that the KD-induced reduction was tissue-specific: the 7-DHC content decreased significantly in the spleen and liver, while the muscle remained unaffected. Sex had no significant influence. Similarly significant main effects of organ and diet on 8-DHC levels were observed. An organ × diet interaction indicated that the KD reduced the 8-DHC levels across all examined tissues, although the extent of this reduction was organ-dependent (spleen > liver > muscle). Notably, a significant organ × sex interaction suggested that females maintained relatively higher levels in the spleen and liver compared to males. DES concentrations varied significantly by organ and were strongly influenced by diet. An organ × sex interaction was also observed, indicating slight sex-based differences in distribution across tissues.

**Figure 10:**
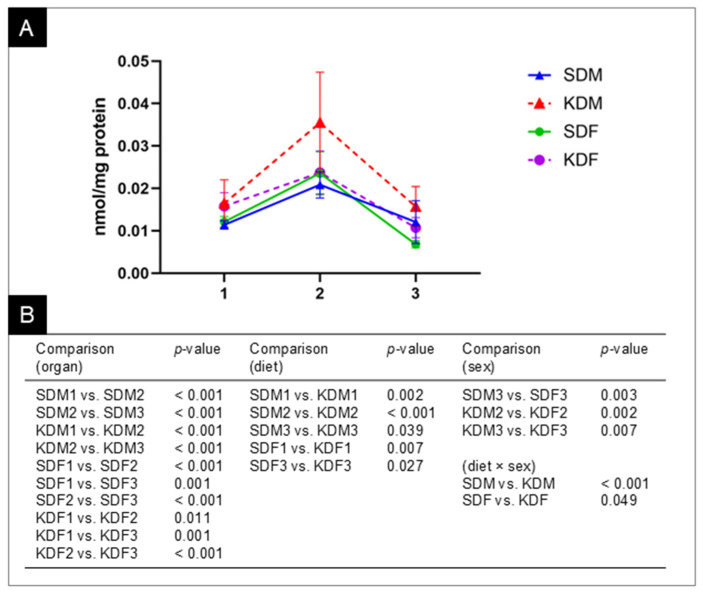
Lanosterol profile analysis in organs (spleen, liver, and skeletal muscle). (**A**) Distribution of lanosterol (LAN) levels in the spleen (1), liver (2), and skeletal muscle (3) of male (M) and female (F) mice on standard (SD) and ketogenic diets (KD). *n* = 10 for all groups, except KDM, where *n* = 9. The graph shows a significant organ × sex × diet interaction, indicating that the metabolic response to the KD varies by tissue type and biological sex. (**B**) Summary of pairwise comparisons. The table presents the statistical significance (*p*-values) for interorgan comparisons, showing significant differences in LAN concentrations across all experimental groups (e.g., SDM1 vs. SDM2); dietary effects, comparing SD vs. KD within specific organ–sex combinations (e.g., SDM2 vs. KDM2); and sex differences, highlighting cases where males and females differ within the same organ and dietary regimen (e.g., KDM2 vs. KDF2). Interaction effects show the overall (diet × sex) impact within the organ dataset. Note: Concentrations are expressed as nmol/mg protein to ensure comparability between tissues. Statistical significance was determined using repeated-measures ANOVA followed by Bonferroni-corrected pairwise comparisons based on estimated marginal means (EMM).

CHOL and LAN exhibited unique profiles, characterized by sex dependency and complex interactions. CHOL showed the highest organ specificity. Notably, diet had no significant main effect on CHOL levels; however, a significant organ × diet interaction indicated that dietary effects were tissue-specific rather than uniform across organs. In contrast, sex was a significant factor, with females generally exhibiting higher concentrations. This was further clarified by a significant organ × sex interaction, which revealed that sex differences were most pronounced in the spleen and liver. As the primary precursor, LAN showed the most complex regulatory pattern. Significant main effects were found for organ, sex, and diet. Furthermore, a significant three-way interaction (organ × sex × diet) was found, along with a significant sex × diet interaction. This indicates that the effect of the KD on LAN levels depends on both the specific organ and the sex of the animal (Figure 10).

## 4. Discussion

KDs are widely used clinically and are popular for weight loss and neurological diseases, yet their long-term systemic metabolic effects, especially on muscles and sterol metabolism, remain incompletely understood. The present study characterized sex-dependent metabolic and sterol adaptations induced by a long-term ketogenic diet (KD) in mice, across the skeletal muscle, liver, spleen, and serum. The results revealed tissue-specific responses that highlighted females’ greater metabolic flexibility during continuous ketosis, characterized by enhanced substrate utilization and reduced sensitivity to adverse lipid remodeling.

### 4.1. Adiposity as the Primary Upstream Driver of Hepatic Lipid Overload

KD induced sex-specific trajectories of body mass gain, with males exhibiting rapid increases beginning at week 1 and females showing a delayed gain from week 3, converging by week 9 despite comparable final increases (~20–25% over baseline). Although adipose tissue depots were not collected for direct quantification, our interpretation that sex-specific adiposity and fat distribution contribute to delayed weight gain in females is based on body weight dynamics and the extensive prior literature on sex-dependent fat accumulation and hormonal regulation under ketogenic and high-fat feeding, rather than on direct adipose mass measurements in this cohort.

Previous studies in C57BL/6J mice consistently report accelerated adiposity in males fed high-fat or ketogenic diets [32], driven in part by testosterone-mediated fat storage and reduced energy expenditure [33]. In contrast, females typically exhibit delayed weight gain linked to estrogen-enhanced lipid oxidation, mitochondrial efficiency, and early metabolic flexibility [34,35]. Estrogen receptor signaling promotes fatty acid β-oxidation and VLDL export while limiting visceral fat expansion, delaying the obesogenic effects of hypercaloric diets until later time points [35]. Thus, in obesity-prone mouse strains and under ad libitum feeding, the KD behaves more similarly to an extreme high-fat diet than a weight loss intervention—an outcome that is unsurprising when caloric intake is unrestricted and dietary fat content is excessive [36]. Age further modulates these effects, as KD exposure has been shown to increase body mass in both young and aged female mice, underscoring the interaction between sex hormones and the maturational stage [37]. Collectively, these findings emphasize that KD-induced adiposity is strongly influenced by sex, hormonal status, and the experimental context.

### 4.2. KD-Induced Hepatic Steatosis and Fibrosis Are Sex-Specific

Consistent with altered body mass trajectories, the KD markedly increased hepatic lipid accumulation, with a significantly larger relative increase in males. Similar degrees of triglyceride accumulation and histological steatosis have been reported in C57BL/6J mice subjected to high-fat or ketogenic feeding, particularly under energy-dense conditions [8,38]. Thus, in terms of hepatic outcomes, the KD closely resembles a steatogenic high-fat diet, with clear sex-dependent differences [21].

Despite the greater relative lipid accumulation in males, females consistently exhibited higher absolute Oil Red O staining across both diets, indicating intrinsically greater baseline hepatic lipid content but improved control over diet-induced progression toward steatosis. While this pattern may reflect the strain-specific characteristics of C57BL/6 mice, it aligns with the broader high-fat diet literature showing that males preferentially direct excess fatty acids to the liver (ectopic fat deposition), whereas females initially buffer lipids in subcutaneous and visceral depots and maintain more efficient hepatic oxidation [38,39]. Estrogen receptor α (ERα) signaling enhances hepatic β-oxidation and VLDL-triglyceride export while suppressing de novo lipogenesis; loss of ERα signaling or ovariectomy abolishes this protection and results in steatosis comparable to that observed in males [40,41]. Conversely, males lack this estrogenic protection and show greater susceptibility to lipotoxicity and inflammatory stress under fat-rich diets, including KDs [21]. KD-specific studies further demonstrate that chronic ketogenic feeding induces steatosis and even fibrosis predominantly in males, whereas females remain relatively protected unless estrogen signaling is disrupted [39,42].

In the present study, the KD increased hepatic lipid droplets by ~43% in males and ~13% in females, consistent with long-term KD studies in both healthy and diabetic mice [43]. Importantly, however, 12 weeks of the KD did not induce hepatic fibrosis in either sex. Picrosirius red staining revealed a preserved extracellular matrix architecture, with a non-significant reduction in collagen density in males and stable levels in females. This contrasts with classical high-fat diet models that promote inflammation-driven fibrosis [44]. While the modest numerical decline in collagen density in males may reflect early antifibrotic remodeling or altered stellate cell activity, the absence of molecular markers precludes mechanistic conclusions [45,46]. Longer-term studies incorporating fibrosis-associated signaling pathways will be required to determine whether prolonged ketosis ultimately promotes matrix remodeling.

Together, these findings indicate that, when administered ad libitum as an unrestricted high-fat regimen, the KD can induce hepatic steatosis in both sexes, with males exhibiting greater vulnerability to lipid overload. At the same time, evidence from other experimental and clinical studies suggests that carefully formulated or time-limited KD protocols can reduce steatosis and improve mitochondrial function in NAFLD, underscoring the strong context dependence of KD effects. Our results therefore contribute to a nuanced body of research, emphasizing sex and dietary context rather than supporting a uniformly harmful or uniformly protective role for the KD.

### 4.3. Ketogenic Diets Alter Skeletal Muscle Metabolism in a Sex-Specific Manner

Spatial metabolomic profiling by MALDI-TOF IMS revealed distinct sex- and diet-specific, tentatively annotated skeletal muscle metabolomic signatures. PLS-DA achieved robust classification accuracy (80–95%) across experimental groups, with network analysis indicating that approximately half of the discriminating *m*/*z* features were group-specific. The most extensive suppression of metabolites converging on acetyl-CoA was observed in standard diet females (SDF), whereas KD-fed groups exhibited more selective alterations.

Tentatively annotated metabolites—including acylcarnitines, inosinic acid, and lysophosphatidylcholines—represent key metabolic entry points into β-oxidation, purine metabolism, and lipid remodeling, respectively. SDF displayed the lowest levels of several of these intermediates, consistent with reduced TCA cycle flux, whereas KD-fed males and females showed targeted reductions in mitochondrial fatty acid transport intermediates. Notably, novel findings included elevated xanthurenic acid 8-O-sulfate in standard diet males and a marked reduction in S-adenosylmethionine (SAM) in KD-fed females—features not previously reported in KD skeletal muscle studies lacking spatial resolution or sex stratification [47].

Functionally, these patterns suggest that the KD shifts muscle metabolism toward ketolysis and lipid-derived energy substrates while reducing glycolytic reliance. However, this reprogramming may come at the cost of mitochondrial overload in males, reflected by acylcarnitine accumulation, and an impaired methylation capacity in females, indicated by reduced SAM levels. Depletion of SAM has been linked to inflammation, oxidative stress, and metabolic dysfunction [48] and may contribute to female-specific susceptibility to muscle atrophy under a prolonged KD via epigenetic dysregulation and suppressed TCA flux. These sex-biased responses align with prior reports of enhanced ketosis and fat oxidation in females [49] and with human KD studies showing substantial reductions in circulating AdoMet despite metabolic improvements [50]. Together, these findings highlight skeletal muscle as a key site of sex-dependent vulnerability during long-term ketogenic adaptation.

### 4.4. Sex-Dimorphic Sterol Regulation in KD

The present study clarifies the tissue-specific and sex-dependent changes in serum and organ sterol profiles induced by the KD, revealing distinct regulatory patterns along the CHOL biosynthetic pathway. Starting from LAN as the initial precursor [51], our findings show that early pathway intermediates respond additively to sex and diet, while late products such as DES and CHOL display strong interactions, highlighting nonlinear metabolic adaptations to the KD.

In serum, the additive effects of sex and diet on early precursors such as LAN and 8-DHC are consistent with their roles as rate-limiting markers of de novo lipogenesis. Males consistently exhibited higher levels across precursors, likely reflecting greater hepatic HMG-CoA reductase activity and androgen-driven sterologenesis, as supported by previous rodent models showing the testosterone-mediated upregulation of squalene monooxygenase [52,53]. The KD elevated LAN independently of sex, consistent with acetyl-CoA carboxylase inhibition and increased mevalonate flux under carbohydrate restriction [54]. Notably, 7-DHC remained diet-insensitive yet sex-dominant (explaining 43.1% of the variance), suggesting that post-LAN enzymes like lathosterol oxidase are regulated by gonadal steroids rather than nutritional modulation [55,56,57]. The pronounced sex and diet interactions for DES and CHOL represent a key novel finding: the KD induced increases in males compared to females. This dimorphism could be explained by the estrogen-mediated suppression of SREBP-2 processing in females, which attenuates KD-driven CHOL accumulation [58,59,60]—a pattern that is reversed in postmenopausal women who exhibit a male-like hypercholesterolemic response in human studies [59,61].

Organ-specific profiles reveal extreme tissue specialization, with the organ type accounting for 76–97% of the sterol variance—a dominant effect that conceals the influence of sex and diet, despite their statistical significance. For example, 97% of CHOL variability arises from interorgan differences alone, reflecting each tissue’s unique biochemical “fingerprint” driven by specialized sterol demands (membranes, steroids, immunity). The KD exerts massive effects on early and mid-precursors (LAN, 8-DHC, 7-DHC > 60% variance explained), as these upstream intermediates act as flexible buffers that are highly sensitive to nutritional changes in acetyl-CoA flux and SREBP signaling [62,63]. Reductions followed a tissue gradient (spleen > liver > muscle for 8-DHC; spleen and liver only for 7-DHC) because the spleen and liver actively manage sterol turnover (immune clearance, bile synthesis), while muscle prioritizes membrane stability during ketosis. Sex effects were minor except for organ and sex interactions, which increased female 8-DHC and DES in the spleen and liver—consistent with estrogen’s modulation of immune and hepatic sterol handling, layered as dominant organ and diet controls [64,65].

Serum and organ sterol regulation are distinctly separated. Organs drastically reduce 7-DHC under the KD (large effect), but serum levels remain stable, indicating that tissues buffer precursors locally without affecting circulation. In contrast, serum CHOL increases significantly under the KD while organ levels remain stable, serving as a circulating reservoir that protects tissue homeostasis. This is most evident for LAN. Serum shows straightforward sex and diet effects, while organs display complex organ, sex, and diet interactions that are not present in the blood.

This pathway progression, from LAN’s multifaceted control to CHOL’s stability, reveals a “funneling” effect—upstream precursors (LAN, 7-DHC, 8-DHC) remain sensitive to diet, organ, and sex, while downstream CHOL is strongly buffered, prioritizing organ homeostasis amid flux. Females generally exhibited higher organ CHOL, likely due to estrogen-enhanced efflux.

These results advance ketogenic metabolism research by quantifying dimorphic, tissue-specific reprogramming, with the discordance between serum and organ findings challenging the reliability of peripheral biomarkers. Sex increased the serum hyperlipidemia risk in males, while the decline in organ precursors may provide an atheroprotective effect. Overall, the KD highlights sex- and tissue-dependent sterol funneling for metabolic resilience.

### 4.5. Study Limitations

This study has several limitations. MALDI-TOF IMS provides a high spatial resolution but relies on tentative metabolite annotations without MS/MS confirmation. Sterol profiling robustly identified sex- and tissue-dependent effects but did not assess underlying regulatory mechanisms at the gene or protein level. Functional measurements of ketolysis, mitochondrial respiration, and muscle performance were not performed, and longer-term outcomes such as fibrosis progression remain unexplored.

## 5. Conclusions

Despite the above limitations, our findings demonstrate that long-term ketogenic feeding induces profound, sex-specific metabolic remodeling across skeletal muscle, liver, and sterol pathways. Females exhibit greater metabolic flexibility but are not fully protected from hepatic lipid accumulation or muscle metabolic suppression. These results underscore the need for the personalized, sex-stratified application of ketogenic diets and highlight skeletal muscle and sterol metabolism as critical determinants of long-term KD outcomes.

## Figures and Tables

**Table 1:** Significant changes in strong *m*/*z* signal intensities in skeletal muscle of standard diet males (SDM), ketogenic diet males (KDM), standard diet females (SDF), and ketogenic diet females (KDF) (*n* = 10 for all groups, except KDM, where *n* = 9), with tentative metabolite annotations. Selection criteria included significant contributions to group separation in the PLS-DA model (variable importance in projection) and significant differences identified in post hoc pairwise comparisons.

*m/z*	Adduct	Treatment Pairs	Tentative Endogenous Metabolite Annotation ^a,b^	Metabolic and Physiological Role	Comments
307.97	M+Na	SDM vs. SDF (↓) SDF vs. KDF (↓) KDM vs. KDF (↓)	Xanthurenic acid 8-*O*-sulfate	Trp/kynurenine metabolism Natriuresis	Xanthurenic acid 8-O-sulfate ^a^ No tentative endogenous hits ^b^
372.24	M+H	SDM vs. KDM (↓) KDM vs. KDF (↓)	Dodecanedioyl carnitine	Beta-oxidation Mitochondrial fatty acid shuttling	No tentative endogenous hits ^a^ Dodecanedioyl carnitine ^b^
387.02	M+K	SDM (↓) vs. SDF SDF vs. KDF (↓) KDM vs. KDF (↓)	Inosinic acid	Purine nucleotide metabolism Neuromodulation, energy regulation	Endogenous tentative hits inosinic acid and inosine 2′-phosphate ^a,b^ Inosinic acid is more likely present in skeletal muscle
399.14	M+H	SDF (↓) vs. KDF	S-Adenosylmethionine	Methylation of DNA, RNA, proteins, phospholipids, and small molecules, polyamine synthesis; methylation status and epigenetic regulation	Tentative endogenous hit ^a,b^
400.34	M+H	SDM vs. SDF (↓) SDF (↓) vs. KDF KDM (↓) vs. KDF	L-Palmitoylcarnitine	β-Oxidation, central intermediate in energy metabolism	Accumulation in tissues under metabolic or ischemic stress ^a,b^
478.33	M+H-H_2_O	SDM vs. SDF (↓)	LysoPC	Cell signaling, lipid metabolism	Endogenous isomers of LysoPC and diacylglycerol ^a^ No tentative endogenous hits ^b^
	M+NH_4_		Diacylglycerol		
496.34	M+H	SDM vs. SDF (↓) SDF (↓) vs. KDF	LysoPC	Membrane remodeling and lipid signaling, pro-inflammatory mediator, lipoprotein metabolism	Two LysoPC isomers ^a,b^
502.33	M+H-H_2_O	SDM vs. SDF (↓) KDM vs. KDF (↓)			

^a^ HMDB [27] search using 50 ppm acceptance limit, ^b^ METASPACE [28] search using 50 ppm acceptance limit. ↓, significantly lowered signal intensity in group; *m*/*z*, mass-to-ratio change. Additional multiple-hit *m*/*z* signals are provided in the Supplementary Materials.

**Table 2:** Statistical analysis of sterol concentrations in serum. The table displays *p*-values, *F* statistics, and partial eta squared (η_p_^2^) values from a two-way ANOVA, assessing the effects of sex, diet, and their interaction on serum sterol levels. *R*^2^ values indicate the proportion of variance explained by the model for each compound.

Factor	LAN	8-DHC	7-DHC	DES	CHOL
Between subjects					
Sex	*p* < 0.001 *F* (1, 35) = 20.1, η_p_^2^ = 0.37	*p* < 0.001, *F* (1, 35) = 28.9, η_p_^2^ = 0.45	*p* < 0.001, *F* (1, 35) = 22.9, η_p_^2^ = 0.4	*p* < 0.001, *F* (1, 35) = 28.9, η_p_^2^ = 0.45	*p* < 0.001, *F* (1, 35) = 26.2, η_p_^2^ = 0.97
Diet	*p* = 0.002, *F* (1, 35) = 10.85, η_p_^2^ = 0.24	*p* < 0.001, *F* (1, 35) = 23.57, η_p_^2^ = 0.4	NS	*p* < 0.001, *F* (1, 35) = 69.2, η_p_^2^ = 0.66	*p* < 0.001, *F* (1, 35) = 249.13, η_p_^2^ = 0.88
Sex × diet	NS	NS	NS	*p* = 0.006, *F* (1, 35) = 8.4, η_p_^2^ = 0.19	*p* = 0.015, *F* (1, 35) = 6.55, η_p_^2^ = 0.16
*R* ^2^	0.48	0.51	0.43	0.75	0.89

NS—not significant; 7-DHC—7-dehydroxycholesterol, 8-dehydroxycholesterol (8-DHC), CHOL—cholesterol, DES—desmosterol (DES), LAN—lanosterol.

**Table 3:** Repeated-measures ANOVA of sterol concentrations across different organs. The table shows the effects of organ (within-subjects factor), sex, and diet (between-subjects factors), as well as their two-way and three-way interactions. Results include *p*-values, *F* statistics with degrees of freedom (adjusted with Greenhouse–Geisser correction where applicable), and the eta squared (η_p_^2^) as a measure of the effect size.

Factor	LAN	8-DHC	7-DHC	DES	CHOL
Between subjects					
Sex	*p* = 0.006, *F* (1, 34) = 8.61, η_p_^2^ = 0.2	NS	NS	NS	*p* = 0.006, *F* (1, 34) = 8.57, η_p_^2^ = 0.2
Diet	*p* < 0.001, *F* (1, 34) = 27.36, η_p_^2^ = 0.45	*p* < 0.001, *F* (1, 34) = 53.89, η_p_^2^ = 0.61	*p* < 0.001, *F* (1, 34) = 83.04, η_p_^2^ = 0.71	*p* < 0.001, *F* (1, 34) = 34.42, η_p_^2^ = 0.5	NS
Sex × diet	*p* = 0.025, *F* (1, 34) = 5.51, η_p_^2^ = 0.14	NS	NS	NS	NS
Within subjects					
Organ	*p* < 0.001, *F* (1.5, 50.5) = 109.64, η_p_^2^ = 0.76	*p* < 0.001, *F* (2, 68) = 211.81, η_p_^2^ = 0.86	*p* < 0.001, *F* (2, 68) = 184.68, η_p_^2^ = 0.84	*p* < 0.001, *F* (2, 68) = 525.68, η_p_^2^ = 0.94	*p* < 0.001, *F* (1.5, 50.5) = 996.93, η_p_^2^ = 0.97
Organ × sex	*p* = 0.043, *F* (1.5, 50.5) = 3.72, η_p_^2^ = 0.1	*p* < 0.001, *F* (2, 68) = 9.12, η_p_^2^ = 0.21	NS	*p* = 0.045, *F* (2, 68) = 3.26, η_p_^2^ = 0.08	*p* = 0.004, *F* (1.5, 50.5) = 7.11, η_p_^2^ = 0.17
Organ × diet	NS	*p* < 0.001, F (2, 68) = 9.52, η_p_^2^ = 0.22	*p* < 0.001, *F* (2, 68) = 30.51, η_p_^2^ = 0.47	NS	*p* = 0.041, *F* (1.5, 50.5) = 3.8, η_p_^2^ = 0.1
Organ × sex × diet	*p* = 0.005, *F* (1.5, 50.5) = 6.94, η_p_^2^ = 0.17	NS	NS	NS	NS

NS—not significant; 7-DHC—7-dehydroxycholesterol, 8-DHC—8-dehydroxycholesterol, CHOL—cholesterol, DES—desmosterol, LAN—lanosterol.

## Data Availability

The raw data supporting this study will be made available by the authors upon reasonable request, as the datasets require expert contextualization to avoid misinterpretation.

## Supplementary Materials

The following supporting information can be downloaded at https://www.mdpi.com/article/10.3390/biomedicines14020462/s1. Figure S1. Percentage change in body mass of male and female mice maintained on a standard diet (SD) or ketogenic diet (KD). Mouse body mass was recorded at baseline (*b*) and weekly thereafter (*w*1–*w*12). Percentage change was calculated as the percentage increase or decrease in body mass relative to the initial baseline value, and it is presented as mean ± SEM. Statistical analysis was performed using the Kruskal–Wallis test (all comparisons *p* < 0.05) followed by Dunn’s multiple-comparisons test. * *p* < 0.05 for SDM vs. KDM; † *p* < 0.05 for SDF vs. KDF. Figure S2. TIC-normalized average mass spectra of selected regions of interest (ROIs) for the comparison of standard diet males (SDM), standard diet females (SDF), ketogenic diet males (KDM), and ketogenic diet females (KDF) within the mass range of 300–700 Da. Figure S3. PLS-DA classification accuracy of skeletal muscle tissue samples into their respective groups within the mass range of 300–700 Da. Figure S4. Box and whisker plots for comparison of tentatively annotated multiple-hit *m*/*z* signal intensities (from 345.21 to 417.05 Da) and across four groups (SDM—blue, KDM—red, SDF—green, and KDF—purple). Figure S5. Box and whisker plots for comparison of tentatively annotated multiple-hit *m*/*z* signal intensities (from 418.05 to 526.33 Da) and across four groups (SDM—blue, KDM—red, SDF—green, and KDF—purple). Table S1. MALDI-TOF metabolite contingency table. Table S2. Significant alterations in the strong *m*/*z* signal intensities of SDM, SDF, KDM, and KDF mouse skeletal muscle with tentative metabolite annotations.
