# Supplementary material for: Sex-Specific Metabolic Footprint of Ketogenic Diet in C57BL/6J Mice
Source: Biomedicines. 2026 Feb 19;14(2):462. doi: 10.3390/biomedicines14020462 (PMC12937886; doi:10.3390/biomedicines14020462)
Supplement: Supplementary file 1 [file biomedicines-14-00462-s001.zip › biomedicines-4104192-supplementary.pdf]

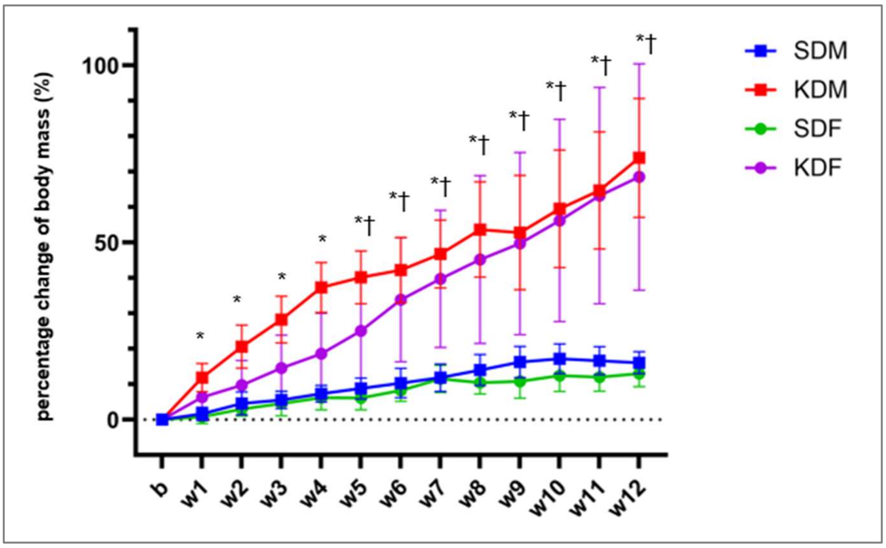

Figure S1. Percentage change in body mass of male and female mice maintained on a standard diet (SD) or ketogenic diet (KD). Mouse body mass was recorded at baseline (b) and weekly thereafter (w1–w12). Percentage change was calculated as the percentage increase or decrease in body mass relative to the initial baseline value, and it is presented as mean ± SEM. Statistical analysis was performed using the Kruskal–Wallis test (all comparisons $p < 0.05$) followed by Dunn's multiple comparisons test. * $p < 0.05$ for SDM vs. KDM; † $p < 0.05$ for SDF vs. KDF.

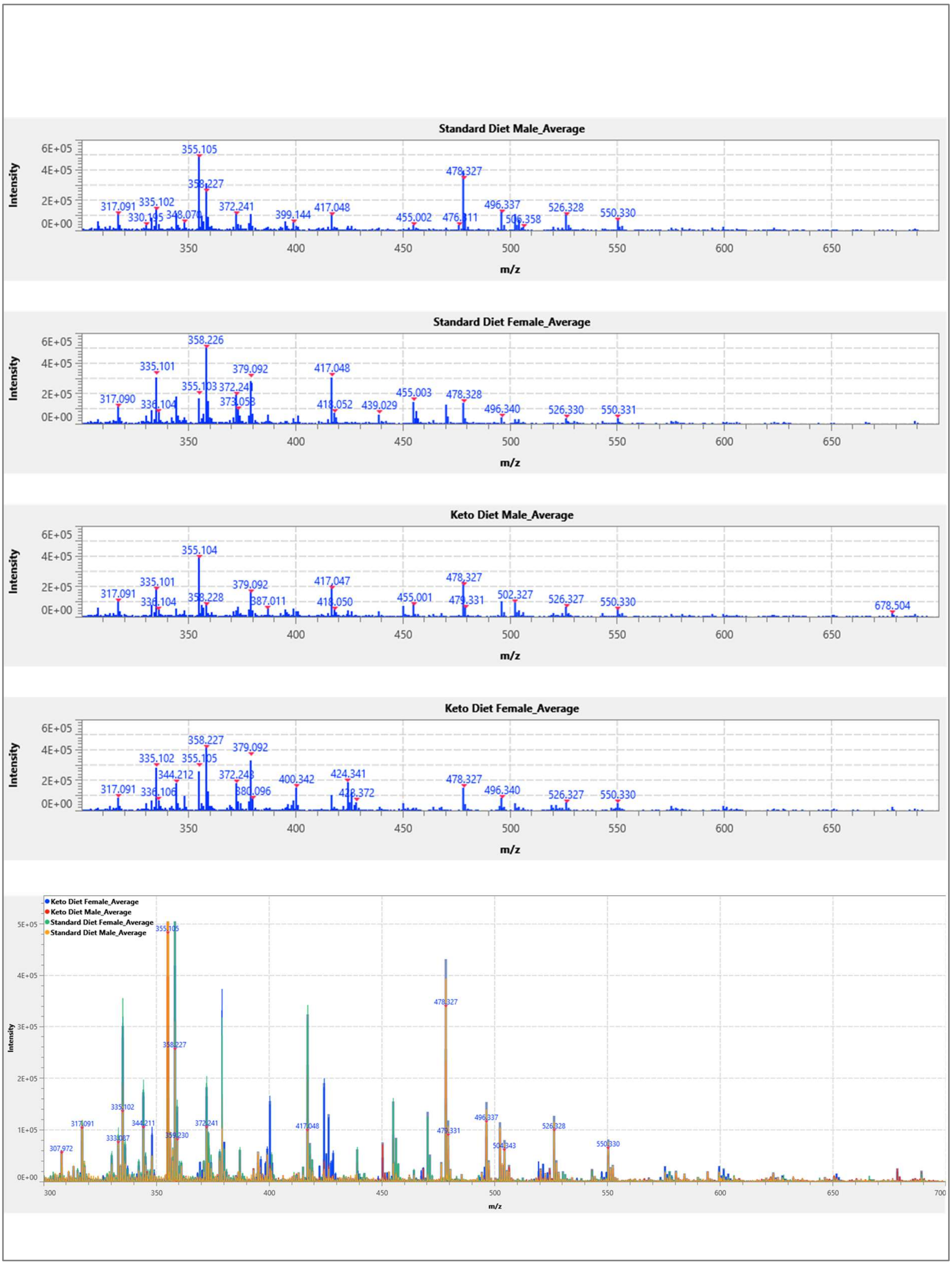

Figure S2. TIC-normalized average mass spectra of selected regions of interest (ROIs) for the comparison of standard diet males (SDM), standard diet females (SDF), ketogenic diet males (KDM), and ketogenic diet females (KDF) within the mass range of 300–700 Da.

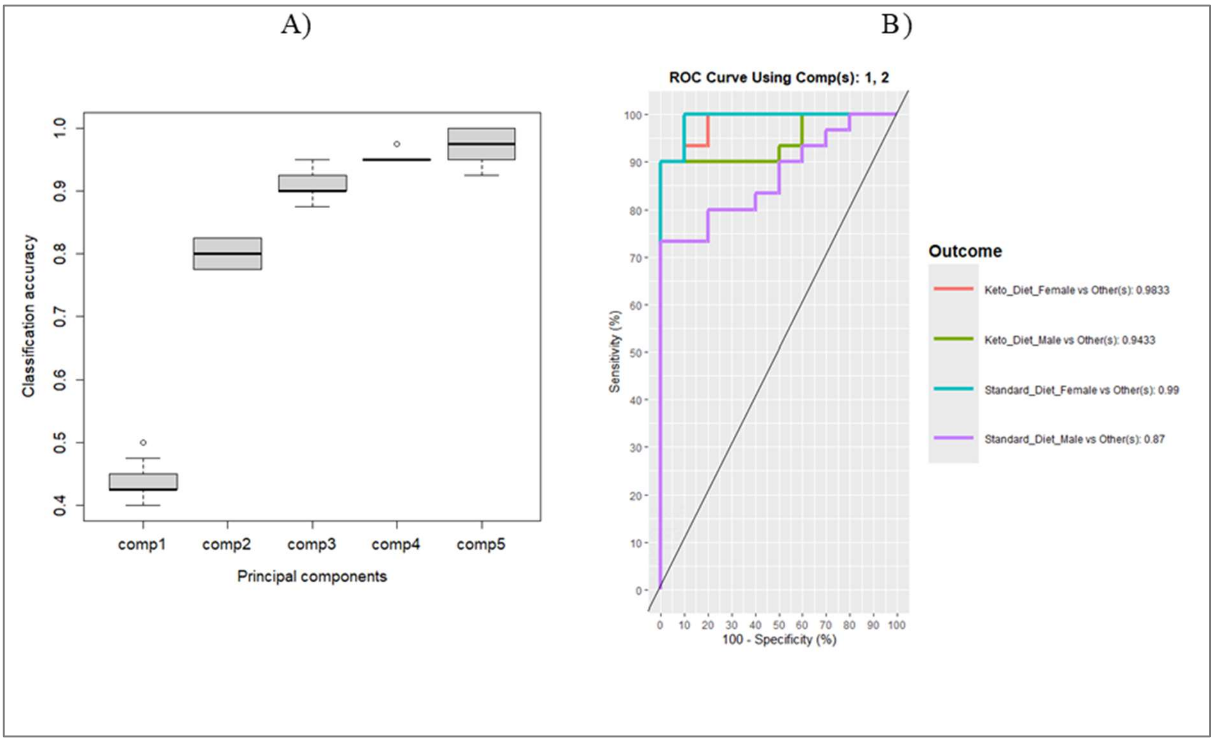

Figure S3. PLS-DA classification accuracy of skeletal muscle tissue samples into their respective groups within the mass range of 300–700 Da. A) Dependence of classification accuracy and number of principal components, with the highest accuracy at 5 principal components (> 95%), B) ROC analysis of the influence of the first two principal components on classification accuracy.

Table S1. MALDI-TOF Metabolites contingency table. The 4×4 contingency table above summarizes all metabolites that were significantly decreased in each treatment group relative to the others. Each row represents the group in which metabolite levels were unchanged or relatively higher, while each column indicates the corresponding group in which those metabolites were significantly lower. Color coding reflects the treatment group in which each metabolite was reduced: blue for KDM, red for KDF, purple for SDM, and green for SDF.

	**KDM**	**KDF**	**SDM**	**SDF**
**KDM**	—	Xanthurenic acid 8-O-sulfate<br>Dodecanedioylcarnitine<br>IMP<br>LysoPC		—
**KDF**	L-Palmitoylcarnitine	—	IMP	S-Adenosylmethionine<br>L-Palmitoylcarnitine<br>LysoPC(0:0/16:0)<br>LysoPC(16:0/0:0)
**SDM**	Dodecanedioylcarnitine	—	—	Xanthurenic acid 8-O-sulfate<br>L-Palmitoylcarnitine<br>Diacylglycerol<br>LysoPC(0:0/16:0)<br>LysoPC(16:0/0:0)<br>Diacylglycerol<br>LysoPC(P-18:1(9Z))<br>LysoPG<br>LysoPS<br>PA
**SDF**	—	Xanthurenic acid 8-O-sulfate<br>IMP	IMP	—

Table S2. Significant alterations in the strong *m/z* signal intensities of SDM, SDF, KDM, and KDF mouse skeletal muscle with tentative metabolite annotations.

m/z	Adduct	Treatment pairs	Tentative endogenous metabolite annotation[a,b]	Metabolic and physiological role	Comments
345.21	M+H-H_2O	SDM vs. KDM (↓)<br>KDM (↓) vs. KDF	*Multiple hits*		Cortisol and 18-Hydroxycorticosterone [a]<br>no tentative endogenous hits[b]
348.07	M+H	SDF (↓) vs. KDF<br>KDM (↓) vs. KDF	*Multiple hits*		adenosine monophosphate (AMP) and similar nucleosides, nucleotides[a,b]
356.11	M+H	SDM vs. SDF (↓)	-	-	no tentative endogenous hits, exogenous: 5-methylthiopentyldesulfoglucosinolate, potential biomarker of soybean oil consumption[a,b]
373.24	*Different types*	SDM vs. KDM (↓)<br>KDM vs. KDF(↓)	*Multiple hits*	-	endogenous MG(0:0/PGJ2/0:0), an oxidized monoacylglycerol involved in lipid signaling[a,b]
380.1	*Different types*	SDM (↓) vs. SDF	*Multiple hits*	-	S-Lactoylglutathione and N6-(delta2-isopentenyl)-adenosine 5'-monophosphate[a]<br>multiple hits[b]
417.05	M+H	SDM (↓) vs. SDF<br>SDF vs. KDF(↓)<br>KDM vs. KDF(↓)	*Multiple hits*	-	Iodothyronine and Monoiodothyronine[a]<br>no tentative endogenous hits[b]
418.05	M+H	SDM (↓) vs. SDF<br>SDF vs. KDF (↓)<br>KDM vs. KDF (↓)	*Multiple hits*	-	Exogenous 5-methylthiopentylglucosinolate, potential biomarker for consumption of coconut oil[a]<br>no tentative endogenous hits[b]
424.34	M+H	SDM vs. SDF (↓)<br>SDF (↓) vs. KDF<br>KDM (↓) vs. KDF	*Multiple hits - carnitines*	Mitochondrial fatty-acid transport for β-oxidation; its presence signals fatty-acid metabolism of trans-unsaturated fats or incomplete β-oxidation	Linoleyl carnitine, 3-Hydroxy-9Z-octadecenoylcarnitine and 3-Hydroxy-11Z-octadecenoylcarnitine[a,b]

<table>
<tr><td>426.36</td><td>*Different types*</td><td>SDM vs. SDF (↓)
KDM (↓) vs. KDF</td><td>*Multiple hits - carnitines*</td><td>-</td><td>N-Linoleoyl Glutamine, 2-Hydroxyhexadecanoylcarnitine, Oleoylcarnitine, Octadecenoylcarnitine, 12-Hydroxy-12-octadecanoylcarnitine, Vaccenyl and Elaidic carnitine[a]
no tentative endogenous hits[b]</td></tr>
<tr><td>450.3</td><td>*Different types*</td><td>SDM (↓) vs. KDM
SDF (↓) vs. KDF</td><td>*Multiple hits*</td><td>-</td><td>N-Eicosapentaenoyl Phenylalanine, N-Arachidonoyl Tyrosine, Heptadecanoylglycerophosphoethanolamine, and LysoPC(14:0/0:0)[a]
Stearoylcarnitine and O-octadecanoyl-R-carnitine[b]</td></tr>
<tr><td rowspan="3">504.34</td><td>$M+H-H_2O$</td><td rowspan="3">SDM vs. SDF (↓)</td><td>LysoPC</td><td rowspan="3"></td><td rowspan="3">endogenous isomers of LysoPC and diacylglycerol[a]
LysoPC(P-18:1(9Z))[b]</td></tr>
<tr><td>$M+NH_4$</td><td>diacylglycerol</td></tr>
<tr><td>M+H</td><td>LysoPC(P-18:1(9Z))</td></tr>
<tr><td rowspan="3">526.33</td><td>$M+H-H_2O$</td><td rowspan="3">SDM vs. SDF (↓)</td><td>LysoPC and LysoPG</td><td rowspan="3"></td><td rowspan="3">endogenous isomers of LysoPC, LysoPG, LysoPS and PA[a]
no tentative endogenous hits[b]</td></tr>
<tr><td>M+H</td><td>LysoPS</td></tr>
<tr><td>$M+NH_4$</td><td>PA</td></tr>
</table>

[a]HMDB [27] search using 50 ppm acceptance limit, [b]METASPACE [28] search using 50 ppm acceptance limit; SDM, standard diet male; SDF, standard diet female; KDM, ketogenic diet male; KDF ketogenic diet female; ↓, statistically significant lowered signal intensity in group; *m*/*z*, mass-to-ratio change

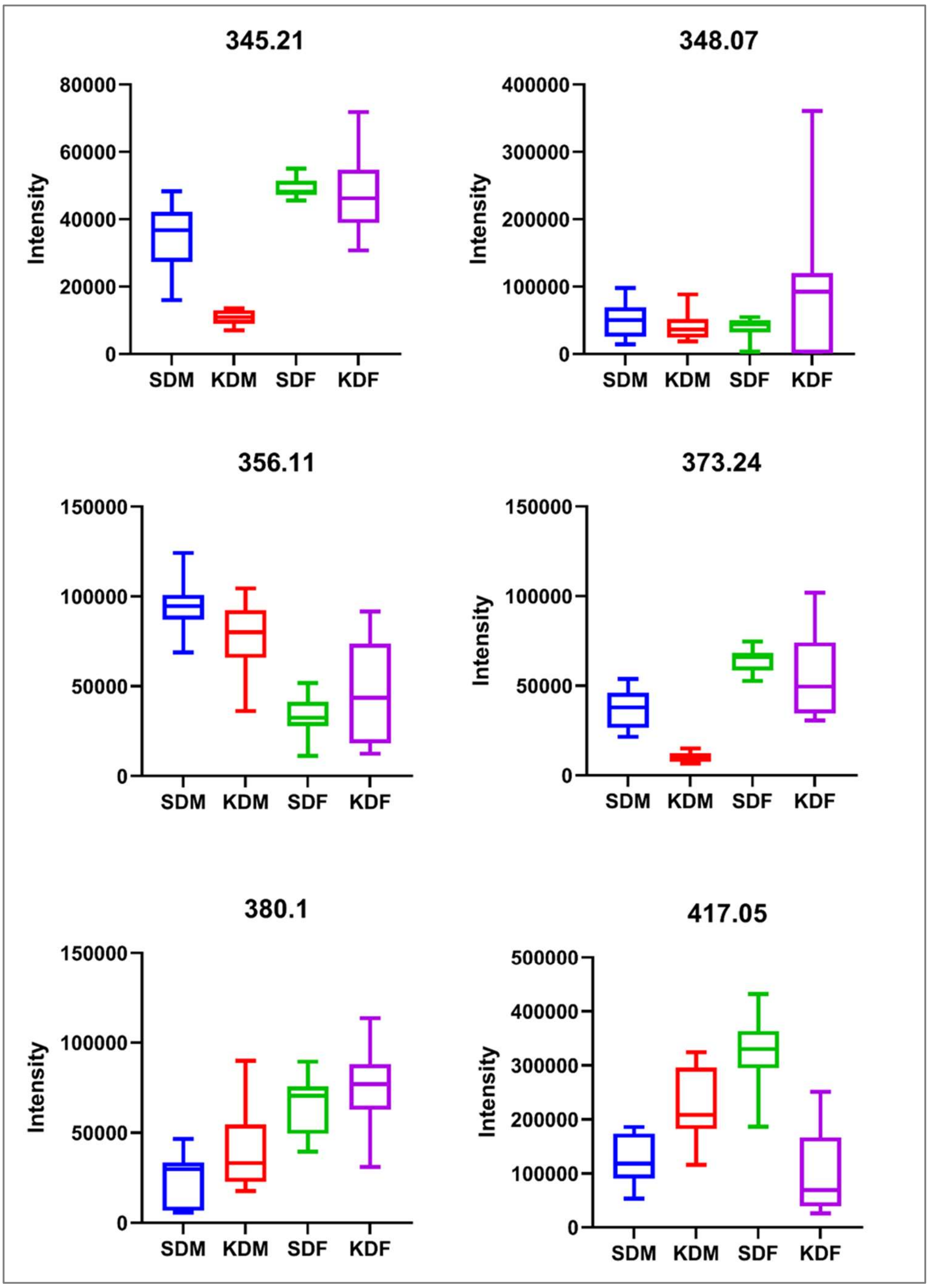

Figure S4. Box and whisker plots for comparison of tentatively annotated multiple hit *m*/*z* signal intensities (from 345.21 to 417.05 Da) and across four groups (SDM — blue, KDM — red, SDF — green, and KDF — purple)

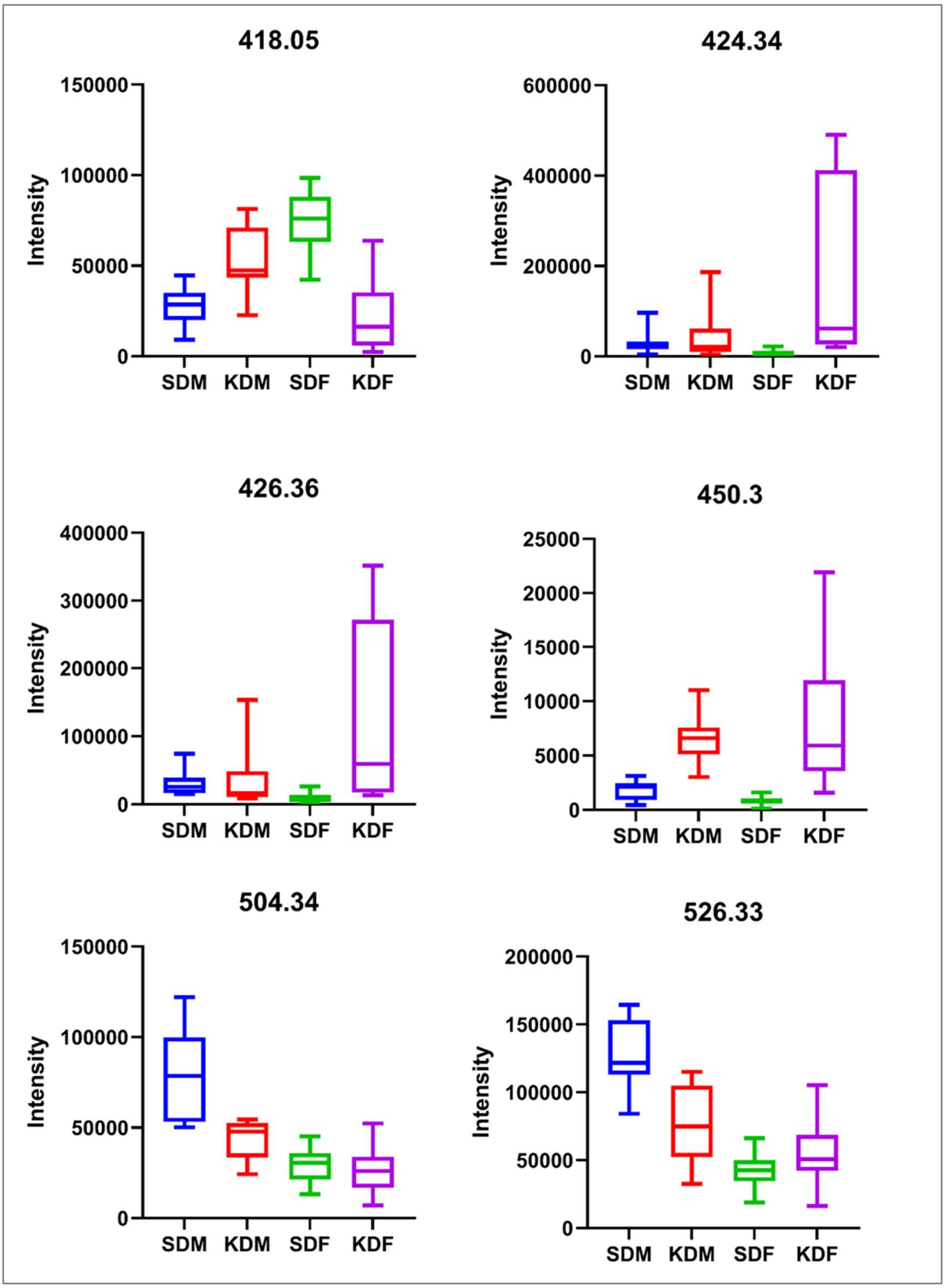

Figure S5. Box and whisker plots for comparison of tentatively annotated multiple hit *m*/*z* signal intensities (from 418.05 to 526.33 Da) and across four groups (SDM — blue, KDM — red, SDF — green, and KDF — purple)
